# 

**Published:** 1857-10

**Authors:** 


### CONTENTS.

### *ORIGINAL COMMUNICATIONS.*

PAGE.
ART. I.—Valedictory Address to the Graduating Class in the Atlanta Medical College. By THOS. S. POWELL, M. D., of Sparta, Ga., ........ 65
ART. II.—Additional Observations upon Veratrum Viride. By V. H. TALIAFERRO, M. D., Atlanta, Ga., ........ 86
ART. III.—Effects of Decayed Teeth. By J. S. DIXON, Ashland, Tenn., .... 89

### *SELECTIONS.*

On the Prevention of Abscess of the Breast by Frictions with the hand, ...... 91
A Case of Loss of nearly half of the Bones of the Skull, with Exposure and Sloughing of a portion of the Brain, ........ 104
Pulmonary Consumption, ........ 108
The Excito-Secretory System, ........ 117

### *EDITORIAL AND MISCELLANEOUS.*

Commencement Exercises, ........ 125
Medical College of Georgia, at Augusta, ........ 125
The Great American Medical Regulator, ........ 126
American Medical Association, ........ 126
Belladonna in Hooping Cough, ........ 127
Bellevue Hospital, ........ 128
Meteorological Gbservations for September, 1857, at Atlanta, Ga. ........ 128

